# Investigation of Transport Processes through Ion-Exchange Membranes Used in the Production of Amines from Their Salts Using Bipolar Electrodialysis

**DOI:** 10.3390/membranes12111126

**Published:** 2022-11-10

**Authors:** Tatyana Karpenko, Nikita Kovalev, Vladislava Shramenko, Nikolay Sheldeshov

**Affiliations:** Physical Chemistry Department, Faculty of Chemistry and High Technologies, Kuban State University, 350040 Krasnodar, Russia

**Keywords:** bipolar membrane, anion-exchange membrane, electrochemical impedance, bipolar electrodialysis, diffusion permeability coefficient, current-voltage characteristics, amines

## Abstract

The influence of the nature of amine solutions on the frequency spectrum of the electrochemical impedance of the bipolar membrane aMB-2m is investigated. Moreover, the effect of the circulation rate of solutions in the electrodialyzer chambers on the volt-ampere characteristics of the Ralex AMH and MA-40L anion-exchange membranes and the aMB-2m bipolar membrane has been investigated. The diffusion characteristics of various types of anion-exchange membranes in a system containing dimethylammonium sulfate ((DEA)_2_H_2_SO_4_), as well as the diffusion characteristics of the Ralex AMH membrane in systems with methylammonium sulfate, dimethylammonium sulfate, diethylammonium sulfate, and ethylenediammonium sulfate ((MA)_2_H_2_SO_4_, (DMA)_2_H_2_SO_4_, (DEA)_2_H_2_SO_4_, EDAH_2_SO_4_) have been studied. It is shown that diffusion permeability depends on the structure and composition of anion-exchange membranes, as well as on the nature of amines. The technical and economic characteristics of the electromembrane processes for the production of amines and sulfuric acid from amine salts are determined. It is shown that when using Ralex AMH anion-exchange membranes in an electrodialyzer together with bipolar aMB-2m membranes, higher concentrations of diethylamine and sulfuric acid are achieved, compared with the use of MA-40L anion-exchange membranes.

## 1. Introduction

Due to the growing demand for the production of organic substances using electromembrane processes, a fundamental understanding of the transport of organic substances in ion-exchange materials is needed. An important issue to study is the question of changing the physicochemical properties of membrane materials due to contact with solutions containing organic substances. Electrodialysis of solutions containing organic ions may differ significantly from the treatment of solutions containing inorganic salts [1,2,3,4].

For the first time, the process of separation of mixtures of weak organic electrolytes by electrodialysis was described in [5]. Investigating systems with a homologous series of alifatic acids, it was observed that the electrical resistance of anion-exchange membranes in the form of carboxylate ions is higher than in sulfate and chloride ones. At the same time, the selectivity of the membranes decreased rapidly with an increase in the size of the acid anion. Dohno R. et al. [6], investigating the electrical conductivity and transfer rates of anion-exchange membranes in solutions of monocarboxylic acid salts, showed a decrease in electrical conductivity from formate to caprinate forms of membranes.

In [7], the electrical conductivity and diffusion permeability of anion-exchange membranes in solutions of sodium hydrothartrate were investigated and it was shown that with a decrease in its concentration, the external electrical conductivity of the membranes increases. The electrical conductivity and diffusion permeability of heterogeneous cation-exchange and anion-exchange membranes in solutions of sodium acetate, acetic, amber, and citric acid were investigated in [8]. The effect on the selectivity and swelling of the anion-exchange membrane of organic acids differing in the number of carboxyl groups (acetic, malonic, and citric), as well as the length of the hydrocarbon radical (formic, acetic, propionic, and *n*-butane) was studied in [9].

The need to obtain amines from their chloride salts to return to the technological cycle arises when obtaining potassium, ammonium and sodium chlorides, potassium and ammonium salts of phosphoric acid as fertilizers, sodium bicarbonate and potassium carbonate salts [10,11,12,13]. At the stage of amine regeneration, calcium hydroxide is used during the alkalinization of their salts, while calcium chloride is formed as a byproduct. Amine regeneration can also be carried out by the electrochemical method in an electrolyzer with a cation-exchange membrane, at the cathode of which the hydroxyl ions formed react with the protonated form of amine, resulting in the formation of amine [14]. In this method, additional chemical reagents other than alkali metal chloride are not used, and the by-products are gaseous hydrogen and chlorine formed on the electrodes.

With the advent of bipolar ion-exchange membranes [15,16,17,18,19,20,21], in which the dissociation of water molecules, in contrast to electrochemical systems, is not accompanied by the release of gaseous products, it became possible to regenerate inorganic and organic acids, alkalis [22,23,24,25,26,27,28], and amines. In these works, the production of pyridine and triethanolamine [29], ethylenediamine [30], ethylene amines [31], dimethylisopropylamine [22], p. 28, ethanolamine [32], piperazine [33,34] in electrodialyzer with a bipolar membrane was investigated. In these devices, one pair of electrodes accounts for orders of magnitude of more elemental cells than when using electrolyzers, as a result of which the consumption of expensive anode materials is sharply reduced.

Despite the abundance of works on the production of acids and alkalis using electrodialysis with bipolar membranes, there are few publications devoted to the production of amines from their salts, and they have not investigated the effect of solutions adjacent to bipolar and anion-exchange membranes on their electrochemical characteristics.

In this paper, the influence of the nature of amines on the overvoltage of the bipolar region of a modified bipolar membrane containing an ion polymer with phosphoric acid groups catalytically active in the dissociation reaction of water molecules, the effect of the circulation rate of adjacent solutions to bipolar and anion-exchange membranes on their current-voltage characteristics, as well as the calculation of technical and economic parameters of the processes of producing amines from their salts using bipolar electrodialysis are investigated.

## 2. Materials and Methods

### 2.1. Objects of the Study

The bipolar membrane under study was an aMB-2m bipolar membrane modified with a phosphoric acid cation-exchange resin, which was synthesized earlier by the authors [35]. The bipolar membrane was synthesized by hot pressing of a Ralex CMH heterogeneous cation-exchange membrane and a Ralex AMH heterogeneous anion-exchange membrane with the predeposition of a phosphoric acid catalyst on the surface of the cation-exchange membrane. Phosphoric acid groups localized in the bipolar region of the membrane [36] accelerate the dissociation of water molecules and decrease the voltage across the bipolar membrane.

Heterogeneous membranes MA-40, MA-40L, MA-41 (Shchekinoazot, sett. Pervomaisky, Russia), and Ralex AMH-PES membrane (Mega, Straz pod Ralskem, Czech) were selected as the anion-exchange membranes under study.

The MA-41 membrane is reinforced with a nylon fabric, and has strong basic quaternary ammonium bases in its composition. Moreover, the Ralex AMH membrane contains strong basic quaternary ammonium bases, but is reinforced with polyestersulfone. The MA-40 and MA-40L membranes contain secondary, tertiary amino groups and quaternary ammonium groups, their difference lies only in the reinforcement fabric: For the MA-40 membrane it is nylon, for the MA-40L it is lavsan.

### 2.2. Investigation of Diffusion Permeability

**Figure 1:**
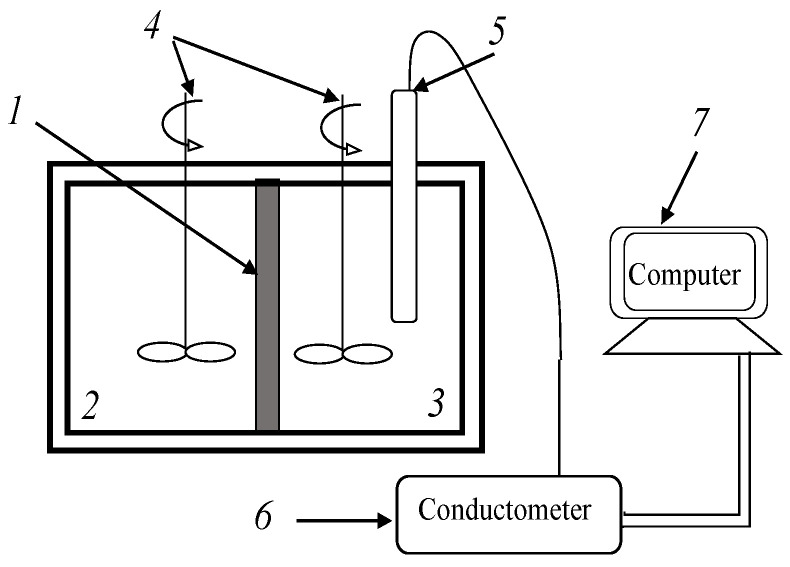
Scheme of a cell for measuring the diffusion permeability of a membrane: *1*—Anion-exchange membrane under study, *2*—chamber with amine salt solution, *3*—chamber with deionized water, *4*—mixers, *5*—conductometric sensor, *6*—conductometer, *7*—computer.

The simplest and most accessible method of studying diffusion permeability [37] is the diffusion of an electrolyte through an ion-exchange membrane into deionized water in a non-flowing two-chamber diffusion cell (Figure 1).

An electrolyte solution (145 mL) was poured into the cell chamber (*2*), deionized water (140 mL) was poured into the cell chamber (*3*), a membrane (*1*) with a working area of 0.19 dm^2^ was located between the solutions. By means of an Expert-002 conductometer connected to a computer, the electrical conductivity of an aqueous solution was measured in chamber (*3*) in time. To eliminate the influence of diffusion layers near the membrane and uniform distribution of the diffusing electrolyte in the solution, the solutions were intensively mixed. Before starting the experiments, all membrane samples were brought into equilibrium with the corresponding 0.5 mol-eq/L electrolyte solutions at 25 ± 1 °C during the 24 h, and then washed with deionized water until the electrical conductivity of the washing waters was constant. The characteristics of diffusion transport were calculated from the experimentally obtained dependence of electrical conductivity on time [37]: Diffusion flux, integral permeability coefficient, β parameter, differential permeability coefficient. An additional study of osmotic water transport through anion-exchange membranes was carried out under conditions of measuring the diffusion permeability of amine salts through them. It was found that the change in the volumes of solutions in chambers 2 and 3 (Figure 1) is very small during diffusion experiments and does not exceed 0.05%; therefore, volume changes were not taken into account when calculating the diffusion permeability coefficients.

Neutral sulfuric acid salts of methylamine ((MA)_2_H_2_SO_4_), dimethylamine ((DMA)_2_H_2_SO_4_), diethylamine ((DEA)_2_H_2_SO_4_), and ethylenediamine (EDAH_2_SO_4_) were used as the studied electrolyte solutions. The concentration of diffusing amine salts was calculated based on the calibration dependencies of their specific electrical conductivity at a temperature of 25 °C. For this purpose, a series of calibration solutions with different concentrations of amine salts in solutions was prepared beforehand and the electrical conductivity of these solutions was measured at a temperature of 25 °C, calibration dependencies were plotted and approximating equations were found.

Furthermore, according to Equation (1), the diffusion fluxes of amine salts through the membrane under study were calculated:

(1) $$j=\frac{V}{S}\frac{dC}{d\tau }$$

where *j* is the diffusion flux density, mol/(m^2^·s); *V* is the volume of water in the chamber into which diffusion occurs, m^3^; *C* is the concentration of electrolyte, mol/m^3^; *S* is the working surface area of the membrane under study, m^2^; *τ* is the time, s.

According to the dependence of the diffusion flow on the concentration in bilogarithmic coordinates, the parameter *β* (Equation (2)) was found, characterizing the shape of the concentration profile inside the membrane during electrolyte diffusion [38]:

(2) $$\beta =\frac{d\mathrm{lg}j}{d\mathrm{lg}C}$$

Then, the integral coefficients (Equation (3)) and differential (Equation (4)) coefficients of diffusion permeability were calculated [38]:

(3) $$P_{m}=\frac{jd}{C}$$

(4) $$P^{*}=P_{m}\beta \;$$

where *P*_*m*_ is the integral coefficient of diffusion permeability, m^2^/s; *d* is the membrane thickness, m; P* is the differential coefficient of diffusion permeability, m^2^/s.

The diffusion permeability coefficient is a quantitative characteristic of the ability of a particular substance to penetrate an ion-exchange membrane under the influence of a concentration gradient. The integral coefficient of diffusion permeability is determined experimentally from the diffusion flux of the substance, and the differential coefficient is calculated taking into account the parameter β.

### 2.3. Impedance Spectroscopy

**Figure 2:**
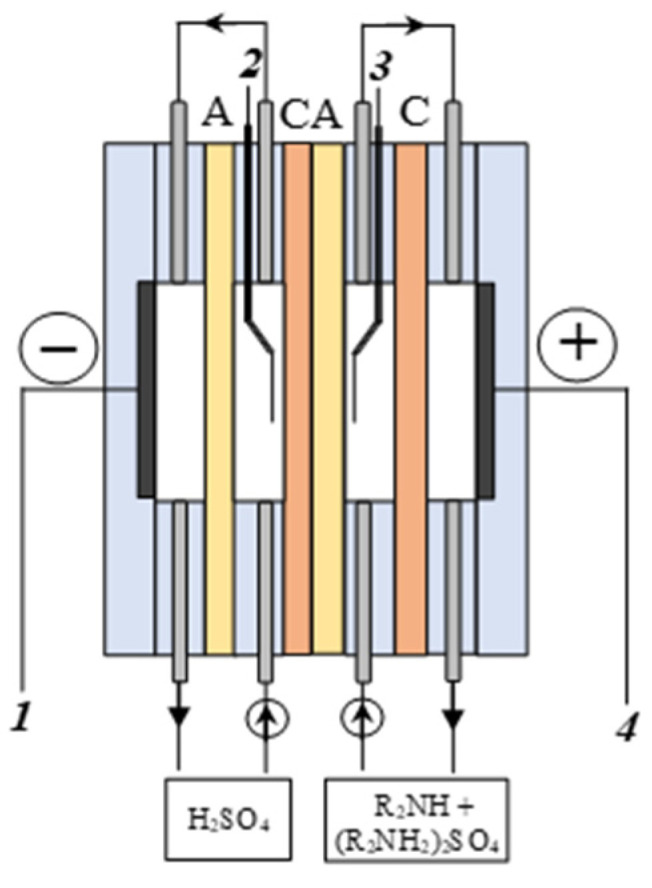
Scheme of an electrochemical cell for measuring the frequency spectra of the electrochemical impedance of a bipolar membrane. C—Ralex CMH cation-exchange membrane; A—Ralex AMH anion-exchange membrane; CA—the studied aMB-2m bipolar membrane; 1, 4—electrodes for the direct and alternating current polarization of the studied bipolar membrane; 2, 3—potential electrodes for measuring the alternating current potential difference across the membrane.

The electrochemical characteristics of the aMB-2m bipolar membrane were studied by impedance spectroscopy in a four-electrode electrochemical cell (Figure 2) as described [39]. Ralex CMH heterogeneous cation exchange membranes and Ralex AMH anion-exchange membranes were used as auxiliary ion exchange membranes to measure the frequency spectra of electrochemical impedance. The measurements were carried out in the «0.5 mol-eq/L sulfuric acid solution | 0.25 mol-eq/L amine and 0.25 mol-eq/L amine sulfate» system, where amine is methylamine (MA), dimethylamine (DMA), diethylamine (DEA), and ethylenediamine (EDA), and the amine sulfate is methylammonium sulfate (MA)_2_H_2_SO_4_, dimethylammonium sulfate (DMA)_2_H_2_SO_4_, diethylammonium sulfate (DEA)_2_H_2_SO_4_, and ethylenediammonium sulfate EDAH_2_SO_4_.

The frequency spectra of electrochemical impedance were obtained using an AUTOLAB 100N potentiostat-galvanostat with a FRA32M frequency impedance measurement module in galvanostatic mode at DC densities from 0 to 2.6 A/dm^2^. The frequency range of the electrochemical impedance spectra was from 0.1 Hz to 1 MHz, measurements were carried out in a stationary mode at a temperature of 25 ± 2 °C. The amplitude of the alternating current voltage drop on the membrane was 50 mV.

**Figure 3:**
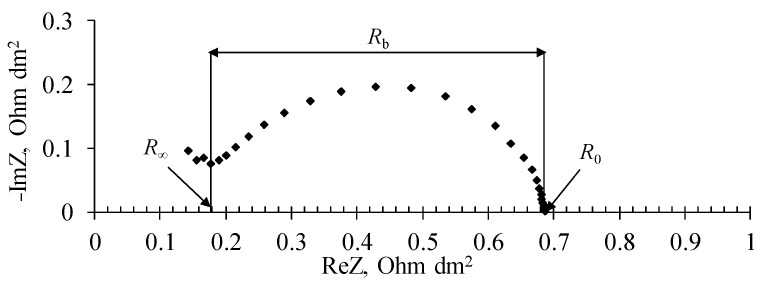
Frequency spectra of electrochemical impedance of bipolar membrane.

The differential resistance of the bipolar region *R*_b_ (Figure 3) was calculated from the frequency spectra of the electrochemical impedance of the membrane according to Equation (5) [26]:

(5) $$R_{b}=R_{0}-R_{\infty }$$

The overvoltage of the bipolar region of the membrane *U*_b_ was found by the dependence of the differential resistance *R*_b_ on the electric current density using Equation (6) [26]:

(6) $$U_{b}=\int_{0}^{i^{*}}R_{b}di$$

where *R*_0_ is the differential resistance, obtained by extrapolating the low frequency part of the impedance spectrum, where the dissociation of water molecules in the bipolar membrane appears, at the Re*Z* axis, Ohm dm^2^ (Figure 3); *R*_∞_ is the differential resistance corresponding to the minimum of the imaginary part of the electrochemical impedance in the high-frequency part of the spectrum, Ohm dm^2^ (Figure 3); *i* is the current density, A/dm^2^.

The calculated overvoltages of the bipolar region were used to obtain a partial current-voltage characteristic of the membrane bipolar region.

### 2.4. IR Spectroscopy

The method of Fourier-transform infrared spectroscopy was used to evaluate changes in the structure of the ion exchange membranes. The IR spectra of the samples of the initial membranes and membranes after use in the electrodialyzer were recorded on the Bruker Vertex-70 device (Ettlingen, Germany) using the module to distribute the total internal reflection in the range of wave numbers 4000–400 cm^–1^.

Preliminary preparation of membrane surface for the analysis consisted in converting all samples into a salt form, holding in 0.5 mol-eq/L sodium chloride solution, washing with deionized water until the electrical conductivity of the washing waters is constant, and drying the samples to an air-dry state. The interpretation of the spectra was carried out using the literature data in [40,41,42].

### 2.5. Current-Voltage Characteristics of the Membrane

**Figure 4:**
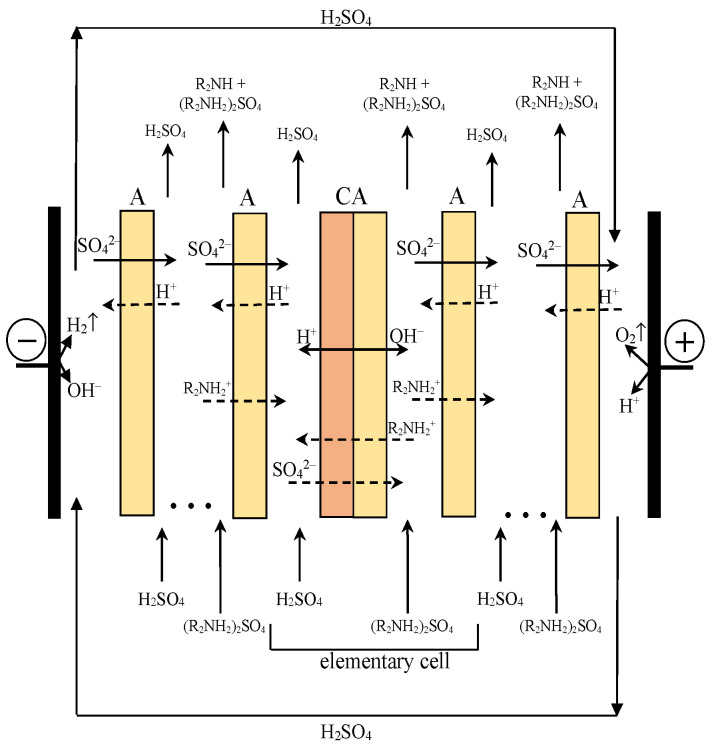
Scheme of a membrane stack of an electrodialyzer designed for the production of diethylamine and sulfuric acid from a diethylammonium sulfate solution. The solid arrows show the fluxes of ions transferred across the membranes, while passing an electric current through the electrodialyzer. The dashed lines show undesirable processes in the membrane stack of the electrodialyzer.

To study the electrochemical characteristics of the Ralex AMH and MA-40L anion-exchange membranes, as well as the aMB-2m bipolar membrane, before and after use in electrodialysis processes for the production of amines from their salts, the dynamic current-voltage characteristics of the membranes were measured at different circulation speeds of a mixture of 0.25 mol-eq/L diethylamine and 0.25 mol-eq/L diethylammonium sulfate and 0.5 mol-eq/L sulfuric acid solution in the chambers of the laboratory electrodialyzer. The study was carried out in the same laboratory electrodialyzer that was used to study the process of amines production (Figure 4). To measure the potential difference across the Ralex AMH and MA-40L anion-exchange membranes and aMB-2m bipolar membranes in measuring dynamic current-voltage characteristics, capillaries connected through a buffer vessel with standard silver chloride electrodes were additionally located on both sides of the studied membranes at an identical distance from the membrane surface.

The measurements were carried out in a galvanodynamic mode in a current density range of 0–3.3 A/dm^2^ at a scan rate of 6.6 × 10^−4^ A/c and a temperature of 25 ± 2 °C, at linear speeds of circulation of solutions equal to 0.5, 1.5, and 2.5 cm/s.

### 2.6. Investigation of Amines Production Processes in Laboratory Electrodialyzer

To study the processes of amines production in a laboratory electrodialyzer, sulfuric acid salts of methylamine, dimethylamine, diethylamine, and ethylenediamine were obtained.

The membrane stack of the electrodialyzer contained five two-compartment unit cells. Each unit cell of the electrodialyzer comprised an acid compartment and a base–salt compartment formed by sequentially located aMB-2m bipolar membranes and Ralex AMH or MA-40L anion-exchange membranes. The choice of this configuration is attributed to the low degree of dissociation of organic bases. In the case of using a three-compartment unit cell, this factor leads to a low electrical conductivity of the solution in the base compartment and the high energy consumption of the process [23]. In the case of using a two-compartment cell, the simultaneous presence of a strong dissociating amine salt and the resulting amine leads to an increase in the electrical conductivity and a decrease in the voltage across the electrodialyzer, which makes it possible to reduce the energy consumption of the process [26].

**Figure 5:**
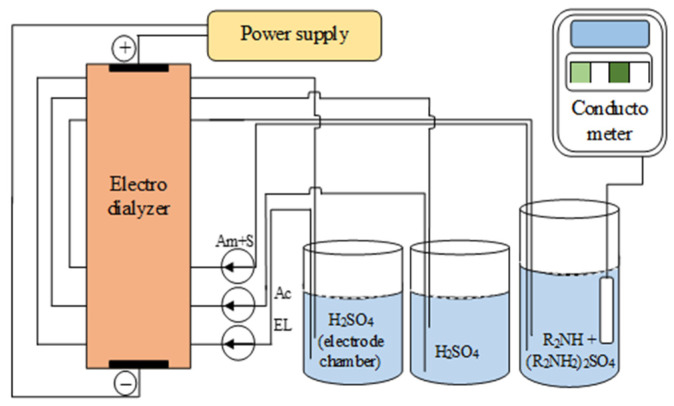
Hydraulic circuit of an electrodialyzer designed to produce amine and sulfuric acid from an amine salt. EL is the electrode chamber solution cycle, Am + S is the base-salt solution cycle, Ac is the acid solution cycle.

The working area of each membrane and electrode was 9 cm^2^; the distance between the membranes of 1.2 mm was set by the thickness of the frames and mesh spacers. The hydraulic circuit (Figure 5) included LOIP LS-301 (Saint Petersburg, Russia) peristaltic pumps, which provided an independent circulation of solutions through the electrodialyzer compartments of the given rates. The linear circulation rate of solutions through each compartment was 2.5 cm/s.

In each hydraulic cycle, both in the measurement of dynamic current-voltage characteristics and in the study of electrodialysis processes, a graduated glass cylinder was used to measure the volume of the solution circulating through each compartment (volumes of solutions in each compartment and tubes through which the solutions were supplied to the electrochemical cell and removed from it were also taken into account).

The electrodialysis process of producing amines and sulfuric acid from sulfuric acid salts of amines was carried out in galvanostatic mode at a current density equal to 2 A/dm^2^. The initial amine sulfate concentration in the salt solution was about 0.5 mol-eq/L; the sulfuric acid concentration was about 0.1 mol-eq/L. At the beginning of the experiment, the temperature of the salt–amine and acid solutions was 25 °C; by the end of the experiment, the temperature increased to 29–30 °C.

At certain intervals throughout the experiment, samples from vessels with base-salt and acid solutions were obtained for subsequent analysis.

The concentration of amine and amine salt in the samples of the solution of the base-salt chamber and the concentration of sulfuric acid and amine salt in the samples of the solution of the acid chamber were determined by titration using an automatic titrator Titroline 6000. The amine concentration was determined by direct titration of the aliquot with 0.1 hydrochloric acid solution. To determine the concentration of the amine salt in the base-salt chamber, an excess of a sample of swollen anionite AB-17-8 in hydroxyl form washed with deionized water was introduced into the sample of a solution of sulfuric acid containing an amine and amine salt and remained under stirring on a magnetic stirrer until the specific electrical conductivity of the solution, measured using a conductometer Expert-002, was constant in time. At this stage, the sulfuric acid was completely removed from the sample and the amine cation was converted to amine. The concentration of the amine in the sample obtained in this way was determined by direct titration with 0.1 mol-eq/L hydrochloric acid solution. Furthermore, the difference was used to find the volume of the titrant used for salt titration, and the concentration of the amine salt was calculated.

The concentration of sulfuric acid in the acid chamber was determined by direct titration of the aliquot with 0.1 mol-eq/L sodium hydroxide solution. The concentration of the amine salt in the acid solution was determined similarly to the concentration of the amine salt in the base-salt chamber.

The time dependencies of the amounts of sulfuric acid and amines in solutions circulating through the compartments of the laboratory electrodialyzer were used to calculate the main technical and economic characteristics of the device in the process of amines and sulfuric acid production from salts.

The integral current efficiencies of sulfuric acid and diethylamine in the electrodialyzer *η* were calculated using Equation (7) [43]:

(7) $$\eta =\frac{F}{IN}\left(\frac{n}{\tau }\right)$$

where *I* is the current supplied to the electrodialyzer, A; *N* is the number of unit cells in the membrane stack of the electrodialyzer; *n* is the amount of the resulting acid or amine, mol-eq; *τ* is the time elapsed since the beginning of the experiment, s; and *F* is the Faraday constant, 96485 (A·s)/mol.

The integral energy consumption required for the production of acid and amine (*E*, kWh/mol-eq) was calculated by Equation (8) [43]:

(8) $$E=\frac{\left(U_{1}-U_{2}\right)F}{N\eta }$$

where *U*_1_ is the voltage supplied to the electrodialyzer, V; *U*_2_ is the voltage supplied to the electrodialyzer containing electrode chambers separated by an anion-exchange membrane at the same electric current at which *U*_1_ was measured, V.

The integral productivity of the electrodialyzer with respect to sulfuric acid and diethylamine (*P*, mol-eq/(h·m^2^)) was calculated by Equation (9) [43]:

(9) $$P=\frac{\eta I}{FS}$$

where *S* is the active area of each membrane, m^2^.

## 3. Results and Discussion

### 3.1. Investigation of Diffusion Permeability

**Figure 6:**
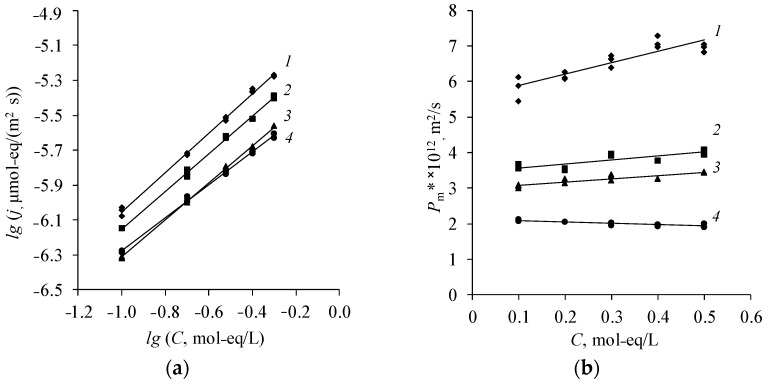
Concentration dependence of the diffusion flux in bilogarithmic coordinates (**a**) and the differential coefficient of diffusion permeability (**b**) of diethylammonium sulfate of various types of anion-exchange membranes. *1*—MA-40L, *2*—MA-41, *3*—Ralex AMH, *4*—MA-40.

**Figure 7:**
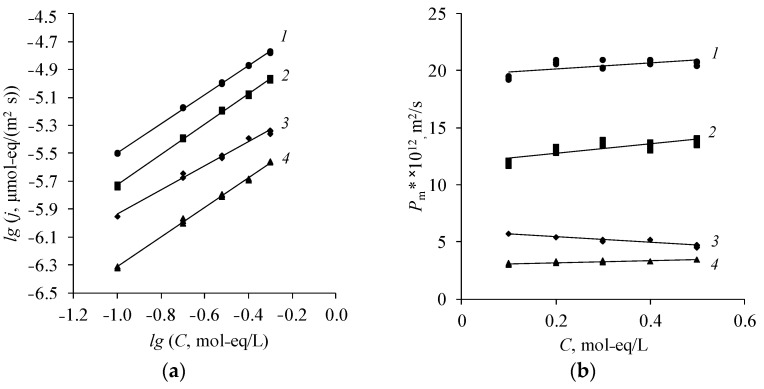
Concentration dependence of the diffusion flux in bilogarithmic coordinates (**a**) and the differential coefficient of diffusion permeability (**b**) of amine salts through the anion-exchange membrane Ralex AMH. *1*—(MA)_2_H_2_SO_4_, *2*—(DMA)_2_H_2_SO_4_, *3*—EDAH_2_SO_4_, *4*—(DEA)_2_H_2_SO_4_.

It is shown that the differential coefficient of diffusion permeability weakly depend on the concentration when studying the diffusion permeability of various natures of amine salts through different types of anion-exchange membranes (Figure 6 and Figure 7). This is explained by the low Donnan sorption of amine cations in the gel phase of the membrane. Therefore, the diffusion of amine salts occurs mainly through the pores of the membrane and the differential coefficient of diffusion permeability remains approximately constant.

The MA-40L membrane has the greatest diffusion permeability (Figure 6). This is due to the fact that the lavsan reinforcement rigidly fixes the membrane; therefore, when it swells, a large number of macropores are formed, through which the diffusion transport of diethylammonium sulfate occurs. The membrane MA-40 has the lowest permeability to diethylammonium sulfate. Nylon is more elastic than lavsan, thus the fraction of pores does not increase with the swelling of the MA-40 membrane. Moreover, the MA-40 membrane has the highest exchange capacity among the presented membranes [8,44]. There is a greater Donnan exclusion of cations (cations of amines) from the gel phase of the membrane under study with an increase in the exchange capacity of the membrane, which in turn prevents the diffusion flux.

The diffusion transport of amine salts of various natures decreases with an increase in their molar mass. Therefore, the salt of the simplest representative of aliphatic amines—methylamine—diffuses through the anion-exchange membrane about five times better than diethylammonium sulfate (Figure 7).

### 3.2. Impedance Spectroscopy

**Figure 8:**
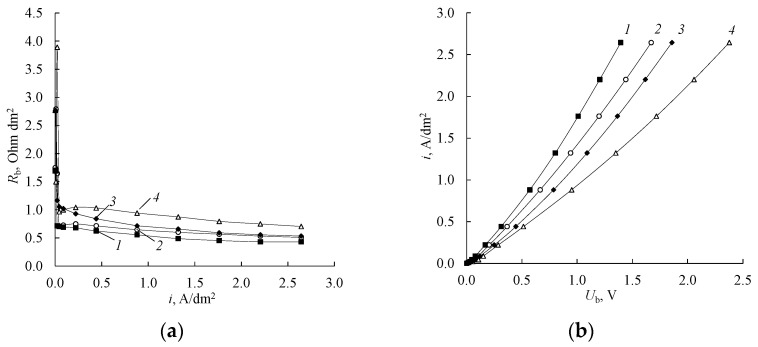
The dependence of the resistance of the bipolar region of the membrane on the current density (**a**) and the partial overvoltage current-voltage characteristics of the bipolar region (**b**) in systems «0.5 mol-eq/L H_2_SO_4_—0.25 mol-eq/L amine, 0.25 mol-eq/L amine sulfate». *1*—DMA, (DMA)_2_H_2_SO_4_, *2*—DEA, (DEA)_2_H_2_SO_4_, *3*—EDA, EDAH_2_SO_4_, *4*—MA, (MA)_2_H_2_SO_4_.

To assess the influence of the nature of amines and the range of current densities in which the bipolar membrane aMB-2m can be used in electrodialysis processes, the dependencies of the resistance of the bipolar membrane region on the current density and the partial overvoltage current-voltage characteristics of the bipolar region in systems with different amines were calculated (MA, DMA, DEA, EDA). It can be observed from the dependencies (Figure 8) that the resistance of the bipolar region increases with an increase in the molar mass and charge of the amine cation. The concentration of salt ions in the bipolar region of the membrane is significantly less than the concentration of hydrogen and hydroxyl ions at current densities above 0.04 A/dm^2^. The hydrogen and hydroxyl ions determine the resistance of the bipolar region of the membrane, since in this case, they are the main carriers of electric current.

The small value of the current density, at which a maximum is observed on the dependence of the resistance of the bipolar region on the current density, indicates a sufficiently high selectivity of the bipolar membrane aMB-2m in solutions used in the process of producing amines from their salts.

### 3.3. IR Spectroscopy

**Figure 9:**
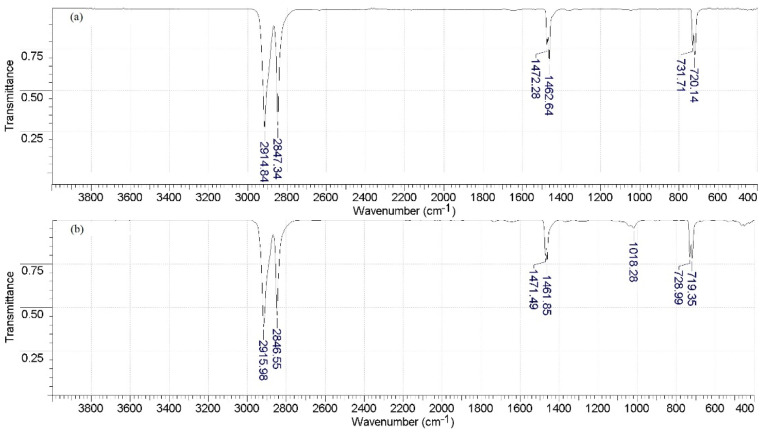
IR spectra of the MA-40L anion-exchange membrane before use in the electrodialyzer (**a**) and after use in the electrodialyzer (**b**).

It can be observed that the MA-40L membranes used in the processes of amine production are characterized by the appearance of peaks in the region of 1018.28 cm^−1^ corresponding to aliphatic amines (C-N bond vibrations) (Figure 9). This may be due to the processes of amine sorption in the membrane phase. Peaks at 2848 and 2915 cm^−1^ in the membranes of MA-40L and Ralex AMH correspond to valence vibrations of CH_2_ methylene groups, and at 1462 and 1472 cm^−1^ to vibrations of the C=C benzene ring in the ion exchange matrix. Peaks in the range of 700–750 cm^−1^ refer to out-of-plane deformation vibrations of the C-H benzene ring.

**Figure 10:**
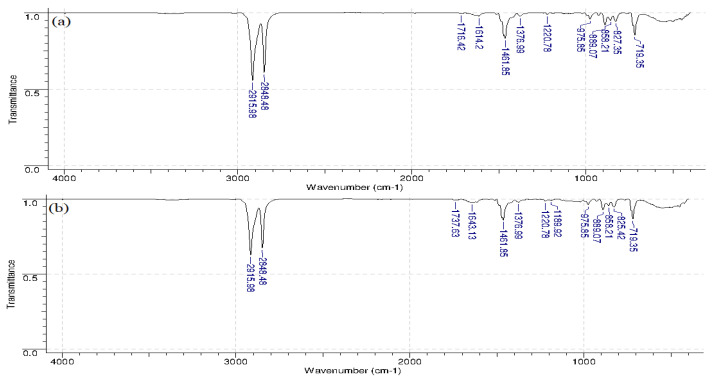
IR spectra of the Ralex AMH anion-exchange membrane before use in the electrodialyzer (**a**) and after use in the electrodialyzer (**b**).

The presence of a peak of 890 cm^−1^ confirms the presence of quaternary ammonium bases in the Ralex AMH membrane –N^+^(CH_3_)_3_ (Figure 10). The displaced peaks on the Ralex AMH membrane before use in the electrodialysis process in the region of 1614 and 1716 cm^−1^, as well as in the membrane after use in the electrodialysis process in the region of 1643 and 1737 cm^−1^ correspond to the deformation vibrations –OH of water molecules.

### 3.4. Voltammetry

**Figure 11:**
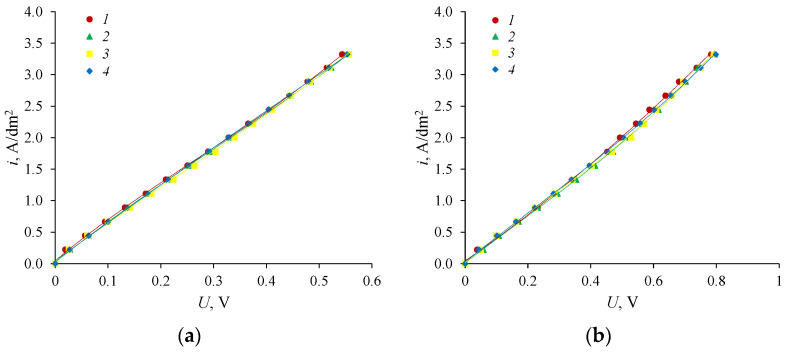
Current-voltage characteristics of the anion-exchange membrane Ralex AMH (**a**) and anion-exchange membrane MA-40L (**b**) at different speeds of circulation of solutions: For initial membrane: *1*—2.5 cm/s, for membrane after use in electrodialysis processes: *2*—0.5 cm/s, *3*—1.5 cm/s, *4*—2.5 cm/s. Each curve was calculated by averaging over the overvoltage of three-time measurements of current-voltage characteristics.

**Figure 12:**
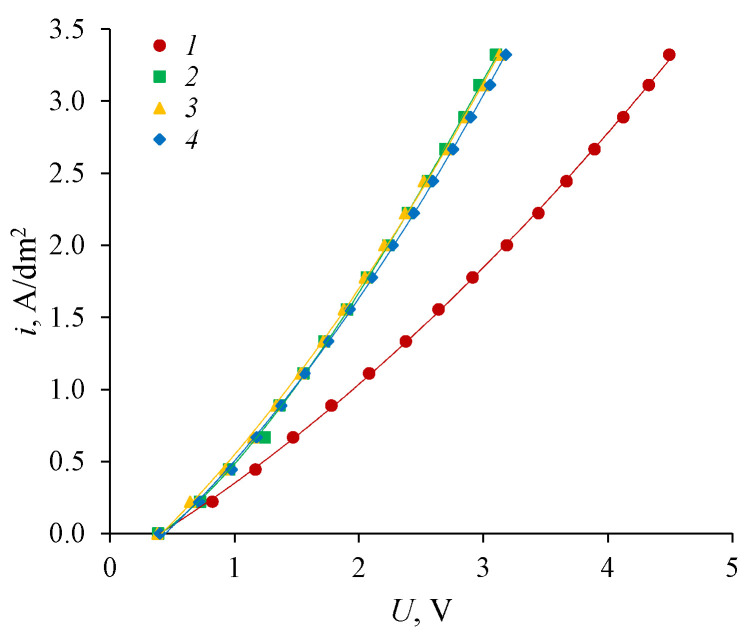
Current-voltage characteristics of the aMB-2m bipolar membrane at different speeds of circulation of solutions: For initial membrane: *1*—2.5 cm/s, for membrane after use in electrodialysis processes: *2*—0.5 cm/s, *3*—1.5 cm/s, *4*—2.5 cm/s. Each curve was calculated by averaging over the overvoltage of three-time measurements of current-voltage characteristics.

To estimate the effect of the circulation rate of amine and sulfuric acid solutions through the chambers of a laboratory electrodialyzer in the electrodialysis process, the current-voltage characteristics of the bipolar membrane aMB-2m and the anion-exchange membranes Ralex AMH and MA-40L were calculated. It is shown that there are no electrodiffusion limiting currents on the membranes in the region of the studied current densities (Figure 11 and Figure 12). This indicates that additional processes typical of currents exceeding the limiting electrodiffusion current, some of which lead to deterioration of the electrochemical characteristics of the membrane, should not develop on the anion-exchange membrane in the area of the studied current densities [45].

It is shown that the circulation rate of solutions adjacent to the anion-exchange membranes under study almost does not affect their current-voltage characteristics. This is due to the fact that the used current densities are significantly lower than the limiting electrodiffusion current (there is no “plateau” of the limiting current) on the membrane. The rate of solutions circulation adjacent to the ion-exchange membranes under study mainly affects the value of the limiting electrodiffusion current and very weakly affects the slope of the current-voltage characteristics of the membranes.

The analysis of the dependencies shows that the circulation rate of solutions does not affect the current-voltage characteristics of bipolar membranes. However, it can be noted that at the same solution speed (2.5 cm/s), the current-voltage characteristics of the bipolar membranes before use in electrodialysis processes for the production of amines (1) and the membranes that were used in electrodialysis processes (2, 3, 4) are markedly different. For bipolar membranes used in amine production processes, there is a decrease in voltage drop compared to the original membranes. This may be due to the breaking of the polyethylene interlayers in the bipolar region during the use of the membrane in the electrodialysis process, which in turn leads to the appearance of new generating contacts in the bipolar region of the membrane [35].

### 3.5. Investigation of Amine Production Processes in Laboratory Electrodialyzer

**Figure 13:**
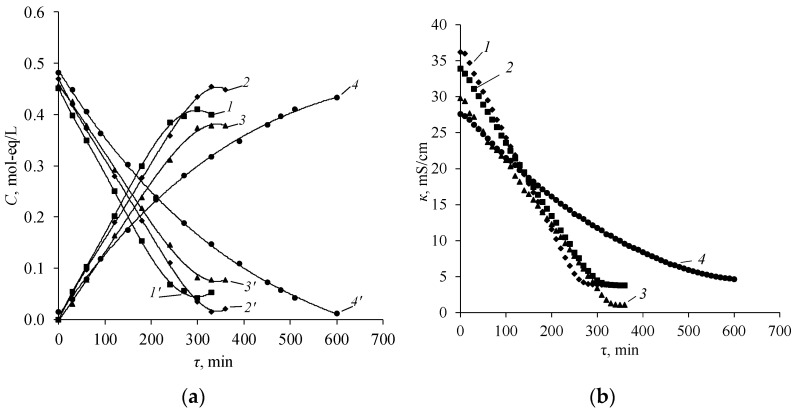
Dependence of the concentrations of sulfuric acid (*1*, *2*, *3*, *4*) and amines salts (*1’*, *2’*, *3’*, *4’*) (**a**) and the specific electrical conductivity (**b**) in the solution circulating through the acid compartments of the electrodialyzer using Ralex AMH anion-exchange membranes on the operating time in the process of production: *1*, *1’*—MA, *2*, *2’*—DMA, *3*, *3’*—EDA, *4*, *4’*—DEA. The solid lines show the approximating curves.

**Figure 14:**
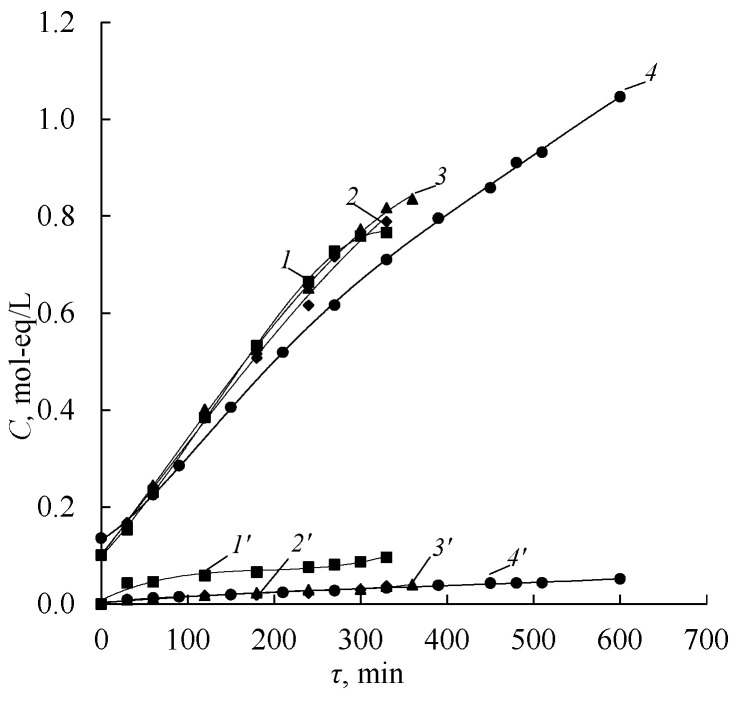
Dependence of the concentrations of sulfuric acid (*1*, *2*, *3*, *4*) and amines salts (*1’*, *2’*, *3’*, *4’*) in the solution circulating through the acid compartments of the electrodialyzer using Ralex AMH anion-exchange membranes on the operating time in the process of production: *1*, *1’*—MA, *2*, *2’*—DMA, *3*, *3’*—EDA, *4*, *4’*—DEA. The solid lines show the approximating curves.

Comparison of the dependencies of the solution concentrations on the operating time of the electrodialyzer with aMB-2m bipolar membranes and Ralex AMH anion-exchange membranes during the process of amine and sulfuric acid production from appropriate salts shows that the concentrations of amines (Figure 13a) and sulfuric acid (Figure 14) increase over time, while the salt concentration decreases (Figure 13a). The completion of the electrodialysis conversion of diethylammonium sulfate to diethylamine is confirmed by the dependence of the electrical conductivity in the base-salt compartment on the operating time of the electrodialyzer (Figure 13b). At all current densities, the specific electrical conductivity decreases and reaches a plateau at a value close to the specific electrical conductivity of a pure amine solution. In the samples of solutions obtained from the base-salt compartment at the times corresponding to the plateau, amine salts are not determined.

Contamination of the produced sulfuric acid solution with amine cations increases with the increasing concentration of sulfuric acid (Figure 14). The presence of salt ions in the acid compartment is caused by the diffusion of the amine through the anion-exchange layer of the bipolar membrane and the diffusion of the amine and amine salt through the anion-exchange membrane into the sulfuric acid solution. These results are consistent with the diffusion permeability studies (Figure 7).

**Figure 15:**
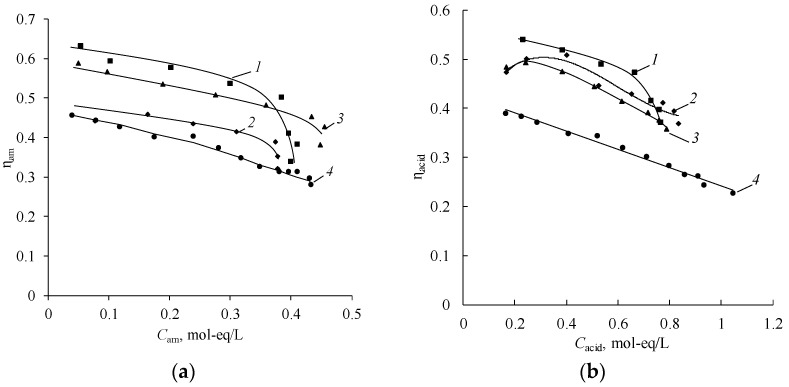
Concentration dependence of the integral current efficiencies of amines (**a**) and sulfuric acid (**b**) in the process of production: *1*—MA, *2*—DMA, *3*—EDA, *4*—DEA. The solid lines show the approximating curves.

The integral current efficiencies of sulfuric acid and amines decrease with an increase in their concentration (Figure 15). In the case of sulfuric acid, the decrease is due to a greater extent of the hydrogen ions leakage from acid to the base-salt chamber across the anion-exchange membrane and to a lesser extent to the migration of the sulfate anion across the bipolar membrane. High effective transport numbers of hydrogen ion through the Ralex AMH anion-exchange membrane in the «DEA, (DEA)_2_H_2_SO_4_–H_2_SO_4_» system are confirmed in [46].

**Figure 16:**
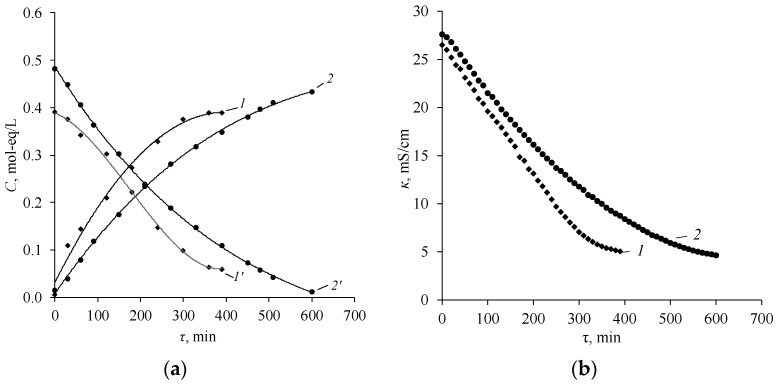
Dependence of the concentrations diethylamine (*1*, *2*) and diethylammonium sulfate (*1’*, *2’*) (**a**) and the specific electrical conductivity (**b**) in the solution circulating through the base-salt compartments of the electrodialyzer on the operating time using as anion-exchange membranes. *1*, *1’*—MA-40L, *2*, *2’*—Ralex AMH. The solid lines show the approximating curves.

**Figure 17:**
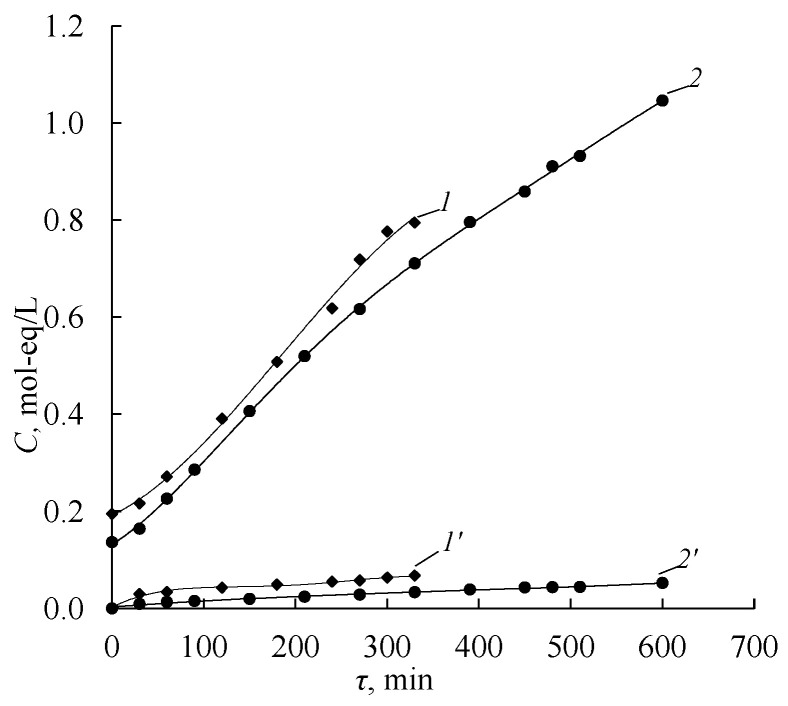
Dependence of the concentrations of sulfuric acid (*1*, *2*) and diethylammonium sulfate (*1’*, *2’*) in the solution circulating through the acid compartments of the electrodialyzer on the operating time using as anion-exchange membranes: *1*, *1’*—MA-40L, *2*, *2’*—Ralex AMH. The solid lines show the approximating curves.

Similar dependencies are observed in the case of the use of the bipolar membrane aMB-2m and the anion-exchange membrane MA-40L in the process of producing diethylamine from diethylammonium sulfate (Figure 16 and Figure 17).

**Figure 18:**
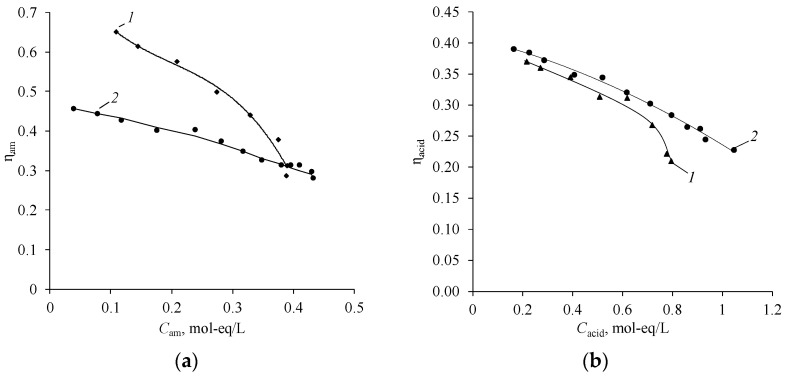
Concentration dependence of the integral current efficiencies of diethylamine (**a**) and sulfuric acid (**b**) used in the electrodialyzer as anion-exchange membranes: *1*—MA-40L, *2*—Ralex AMH. The solid lines show the approximating curves.

However, it is worth noting that the process using MA-40L membranes as anion exchange is more intensive at the initial stages of the process, which is confirmed by an increase in the slope of the dependence of the concentrations of salt, amine, acid, and the electrical conductivity of the base-salt solution on the operating time of the electrodialyzer. At the same time, the current efficiency of diethylamine when using MA-40L membranes drops sharply with an increase in the concentration of the resulting amine (Figure 18).

The main technical and economic parameters of the process are presented in (Table 1). The integral energy consumptions increase with an increase in the concentrations of the produced amines and acid, despite the fact that the voltage on the electrodialyzer slightly decreases during the entire process. The increase in integral energy consumptions is not associated with an increase in voltage due to the decrease in the concentration of amine salt, which has high conductivity, and an increase in the concentration of weakly dissociating amine in the base-salt chamber. The voltage on the entire electrodialyzer is determined by a faster decrease in the voltage on the acid chamber with an increase in the concentration of sulfuric acid in it. A possible cause of the increase in the integral energy consumption may be a decrease in the current efficiency of the amine and acid (Equation (8)) with an increase in their concentrations in solutions. A decrease in the current efficiency (Equation (9)) with an increase in the amine and acid concentrations in solutions causes a decrease in the integral productivity of sulfuric acid and amines (Table 1).

## 4. Conclusions

Despite the high effective transport numbers of hydrogen ion through the anion-exchange membrane under the conditions of the process of producing amines and sulfuric acid from amine salts and not too high current efficiencies of amines and acid, it is possible to achieve almost complete conversion of the amine salt into amine. An additional decrease in the current efficiency of methylamine is caused by its high coefficient of diffusion permeability through the anion-exchange membrane. The use of bipolar aMB-2m membranes and heterogeneous anion-exchange membranes in the conversion process of amine salts into amines makes it possible to produce sufficiently pure sulfuric acid solutions with an equivalent amine fraction not exceeding 12% for methylamine and not exceeding 5% for DMA, DEA, and EDA.

## Figures and Tables

**Table 1:** Characteristics of electrodialysis processes using modified bipolar membrane aMB-2m and anion-exchange membranes Ralex AMH (1–4) and MA-40L (5) at a DC density equal to 2 A/dm^2^.

Process Characteristics	Dimension	Source Salt (0.5 mol-eq/L)				
		1	2	3	4	5
		EDAH_2_SO_4_	(MA)_2_H_2_SO_4_	(DMA)_2_H_2_SO_4_	(DEA)_2_H_2_SO_4_	(DEA)_2_H_2_SO_4_
Operating time of the electrodialyzer	min	360	330	360	600	390
Concentration of sulfuric acid in the acid chamber	mol-eq/L	0.79	0.77	0.84	1.05	0.79
Integral current efficiency of sulfuric acid		0.36	0.37	0.37	0.23	0.21
Integral energy consumption in the production of sulfuric acid	kWh/ mol-eq	0.32	0.20	0.19	0.45	1.2
Integral productivity in the production of sulfuric acid	mol-eq/ (h·m^2^)	2.70	2.84	2.80	1.75	1.62
Amine concentration	mol-eq/L	0.45	0.40	0.38	0.43	0.39
Integral current efficiency of amine		0.38	0.34	0.32	0.28	0.29
Integral energy consumption in the production of amine	kWh/ mol-eq	0.29	0.21	0.22	0.37	0.87
Integral productivity in the production of amine	mol-eq/ (h·m^2^)	2.99	2.67	2.50	2.09	2.22

## Data Availability

Not applicable.

## Abbreviations

MA	methylamine	
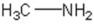
(MA)_2_H_2_SO_4_	methylammonium sulfate	
DMA	dimethylamine	
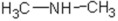
(DMA)_2_H_2_SO_4_	dimethylammonium sulfate	
DEA	diethylamine	
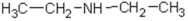
(DEA)_2_H_2_SO_4_	diethylammonium sulfate	
EDA	ethylenediamine	
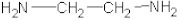
EDAH_2_SO_4_	ethylenediammonium sulfate	
